# Supplementary material for: The gastrointestinal pathogen Campylobacter jejuni metabolizes sugars with potential help from commensal Bacteroides vulgatus
Source: Commun Biol. 2020 Jan 7;3:2. doi: 10.1038/s42003-019-0727-5 (PMC6946681; doi:10.1038/s42003-019-0727-5)
Supplement: Supplementary file 1 — Supplementary Information [file 42003_2019_727_MOESM1_ESM.pdf]

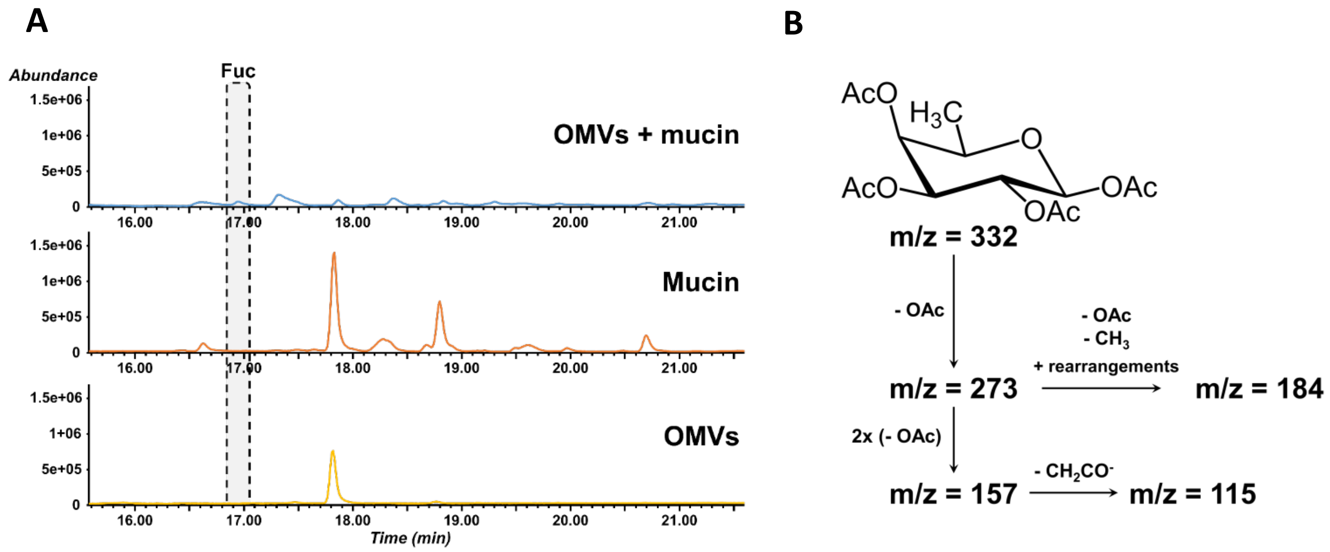

Supplementary Figure 1. Confirmation of fucose release from porcine mucin sample. (**a**) Comparative GC-MS chromatograms from untreated *B. vulgatus* outer membrane vesicles (OMVs), untreated 0.5% porcine mucin (mucin), and 0.5% mucin treated with outer membrane vesicles (OMVs+mucin). Free L-fucose (Fuc) is only evident in the sample containing both OMVs and mucin suggesting *B. vulgatus* fucosidases are required to release the free sugar from mucin. (**b**) Predicted electrospray ionization-mass spectrometry fragmentation pattern of L-fucose with corresponding masses indicated.

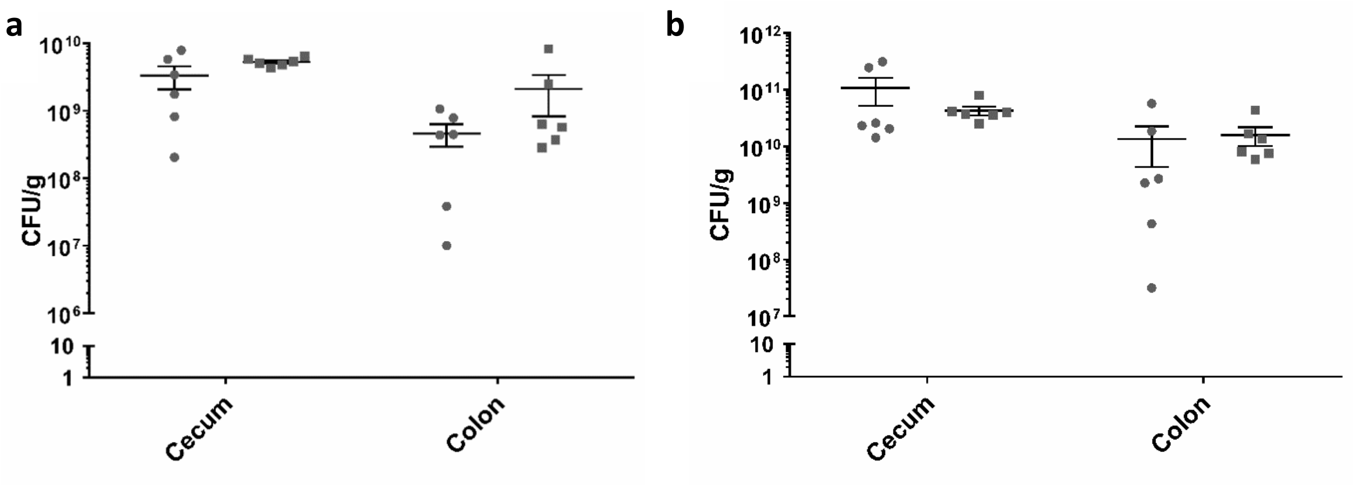

Supplementary Figure 2. ***C. jejuni* (a) and *B. vulgatus* (b) cecal and colon counts as indicated.** Each shape represents colony counts of the bacterium either alone (circles) or in co-culture (squares) from a single mouse. Bars denote means and error bars show standard error of the mean.

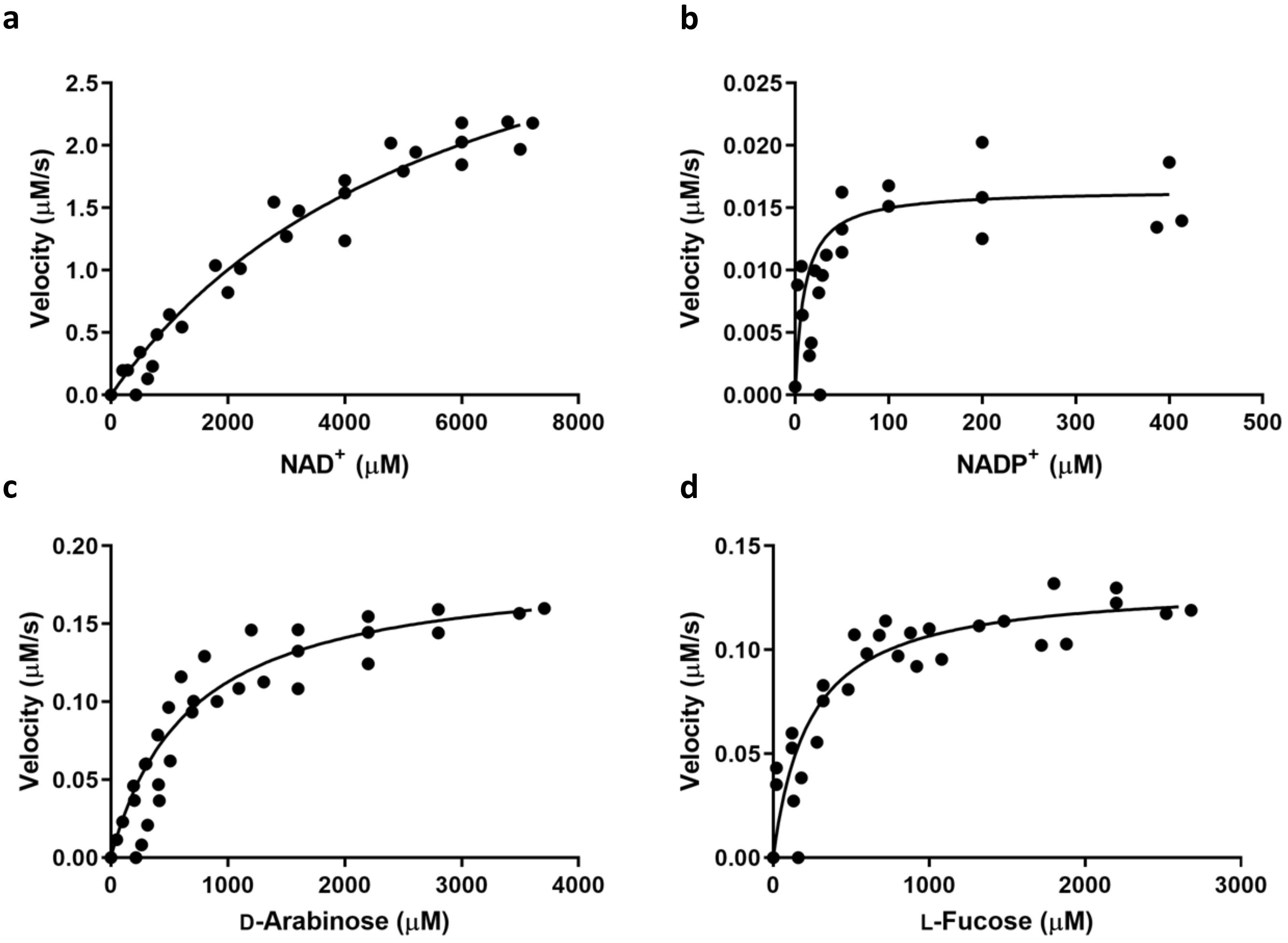

Supplementary Figure 3. Kinetic analysis of FucX with NAD$^+$ (a), NADP$^+$ (b), L-fucose (c) and D-arabinose (d). The units of the y-axis refer to the rate of NADH (**a**) or NADPH (**c-d**) equivalents produced. In the assays, 200, 1, 5 or 10 nM FucX was used respectively for the NAD$^+$, NADP$^+$, L-fucose and D-arabinose results shown in a-d. Individual values from three technical replicates are shown.

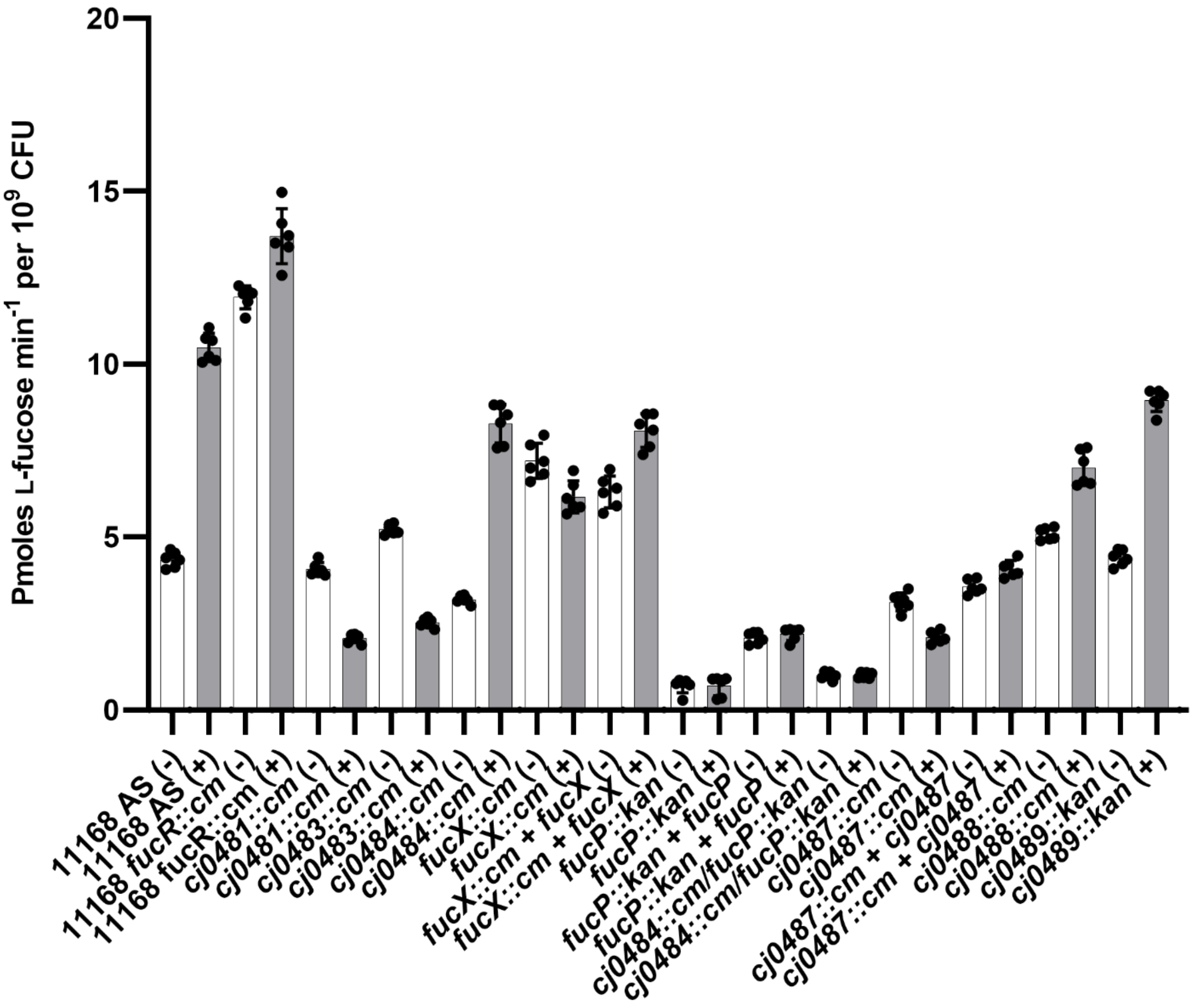

Supplementary Figure 4. L-fucose uptake by *C. jejuni* 11168 wildtype and fucose mutants. ^{3}H-L-fucose uptake rates in cells of the wildtype, the indicated mutants and complemented mutant strains grown in the absence (white) or presence (gray) of 10 mM L-fucose are shown. Each bar represents the mean value obtained from three technical replicates within two independent experiments carried out in duplicate. Standard deviations are indicated by the error bars. Values from individual replicates are overlaid as dots.

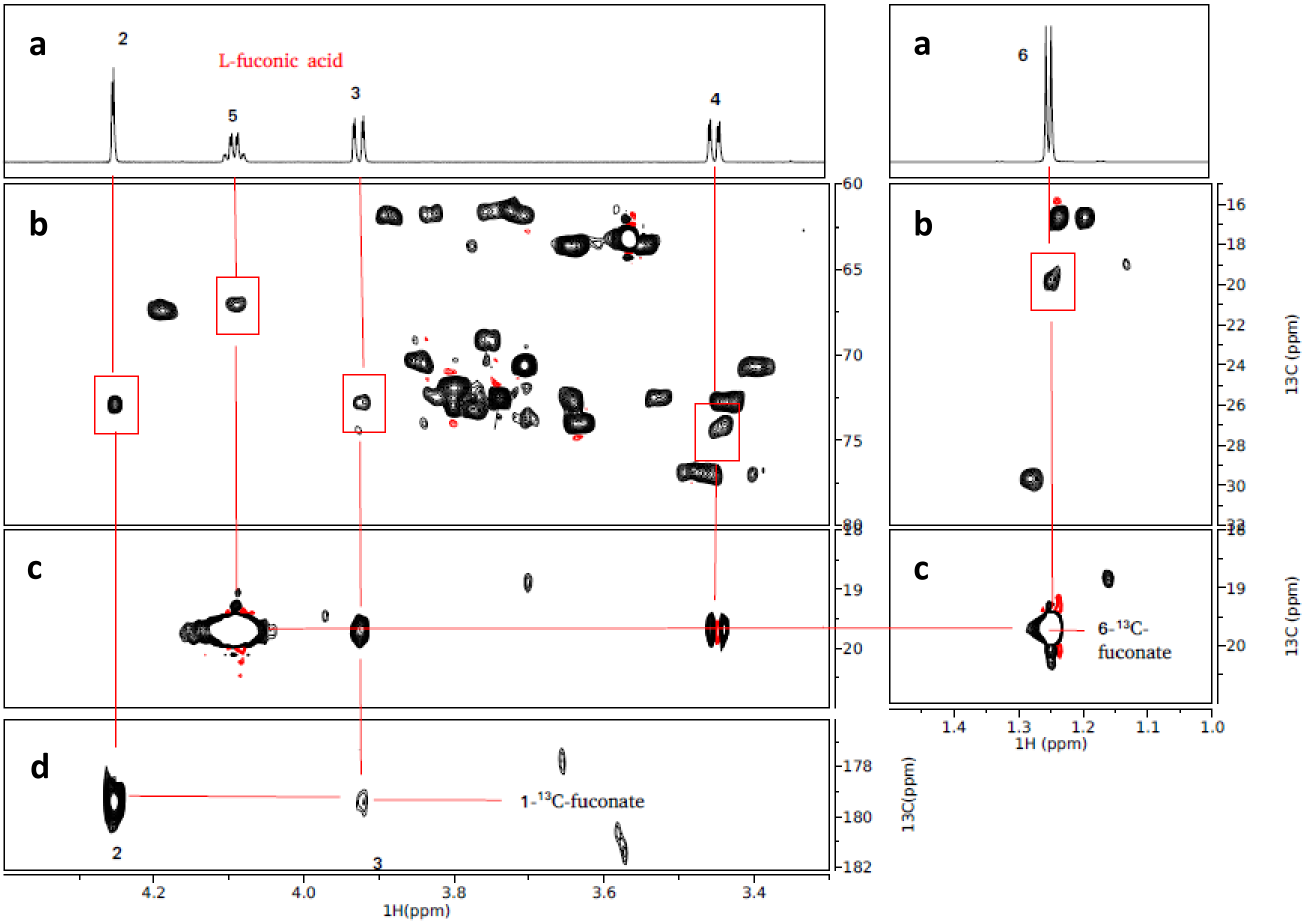

Supplementary Figure 5. Regions of the 800 MHz NMR spectra showing *C. jejuni* 11168 extracts grown on 1-^{13}C-fucose and 6-^{13}C-fucose. (**a**) Proton spectrum of L-fuconic acid. (**b**) ^{13}C,^{1}H-HSQC spectrum showing signals belonging to L-fuconic acid (red boxes). (**c**) ^{13}C,^{1}H-HSQC-TOCSY spectrum showing connectivity between the ^{13}C-labeled C_6 of fuconic acid to other protons. H_2 is not seen in the trace due to the small H_3-H_2 coupling. (**d**) ^{13}C,^{1}H-HMBC spectrum showing the connections between 1-^{13}C-carboxyl and H_2 and H_3 of the fuconic acid.

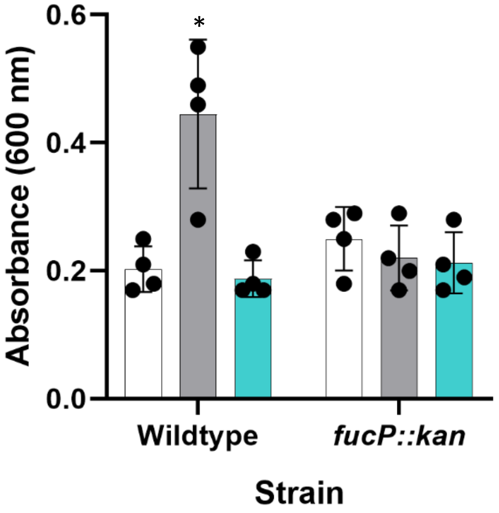

Supplementary Figure 6. **L-galactose does not enhance *C. jejuni* 11168 growth in minimal medium.** Growth in minimal medium either unsupplemented (white), or supplemented with 25 mM L-fucose (gray) or 25 mM L-galactose (cyan) was assessed by optical density readings at 600 nm after 18 hours of microaerobic incubation at 37°C. Values represent means of four biological replicates and error bars represent the standard deviation. Values from individual replicates are overlaid as dots. The asterisk indicates significant growth enhancement in comparison to the unsupplemented control (p=0.022).

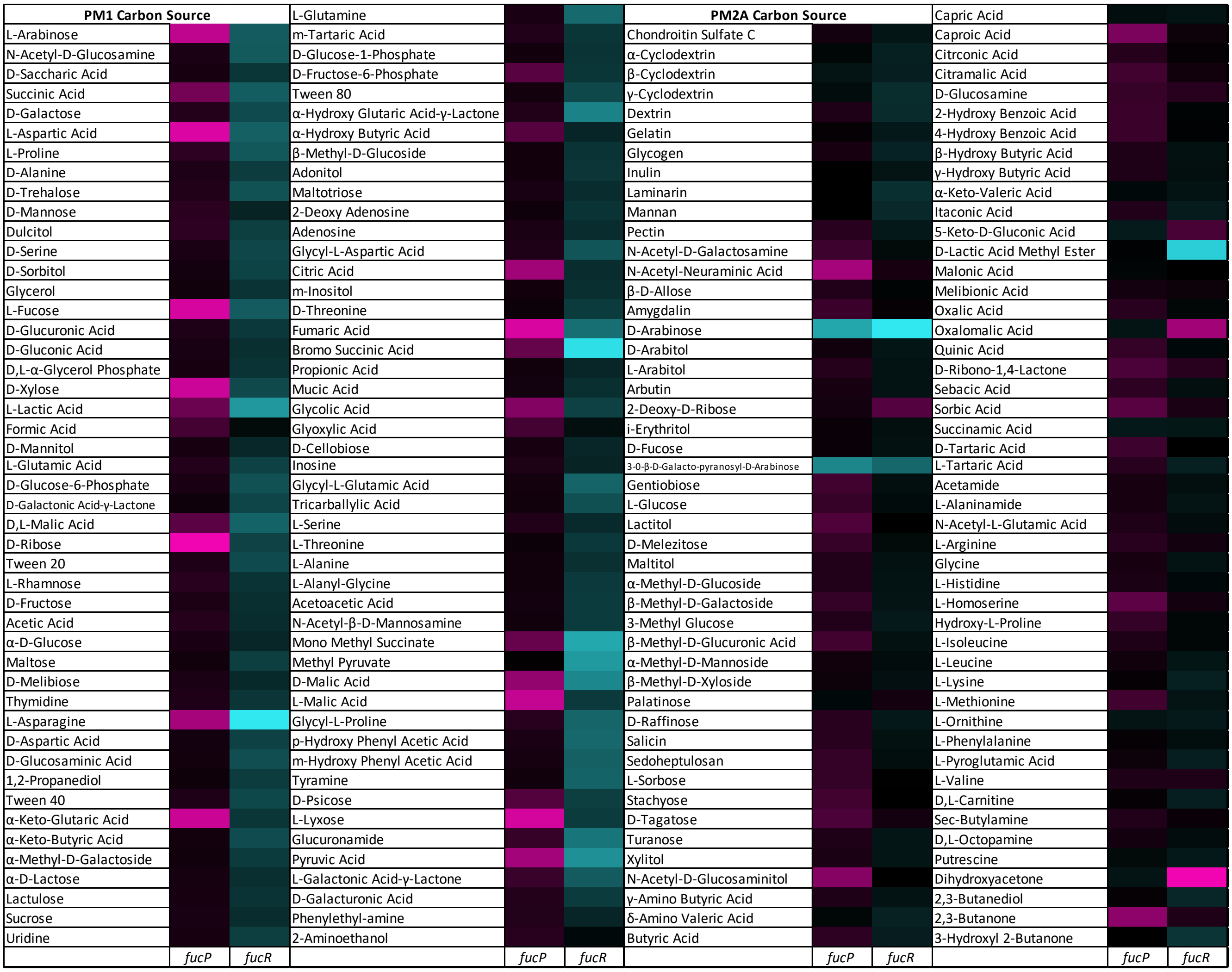

Supplementary Figure 7. **Biolog screening for carbon source utilization by *C. jejuni* 11168 wildtype versus mutants indicated at the bottom of each heat map.** Cells were incubated in PM1 and PM2A (Biolog) phenotypic microarray plates containing common carbon sources under microaerobic conditions at 37 °C. Data are averages of two biological replicates. The heat map shows an average of absorbance differences after 72 hours. A greater increase in dye reduction (indicator of respiration on the carbon source) relative to wildtype is indicated by increased cyan coloration whereas increased magenta coloration indicates decreased respiration. Black coloration indicates no difference between wildtype and mutant respiration. Note that the responses for L-arabinose, D-xylose, D-ribose, L-lyxose, D-arabinose, D-glucosamine, butyric acid, and dihydroxyacetone should be interpreted with caution as these wells gave some reaction using the indicator dye in a negative control plate without cells present.

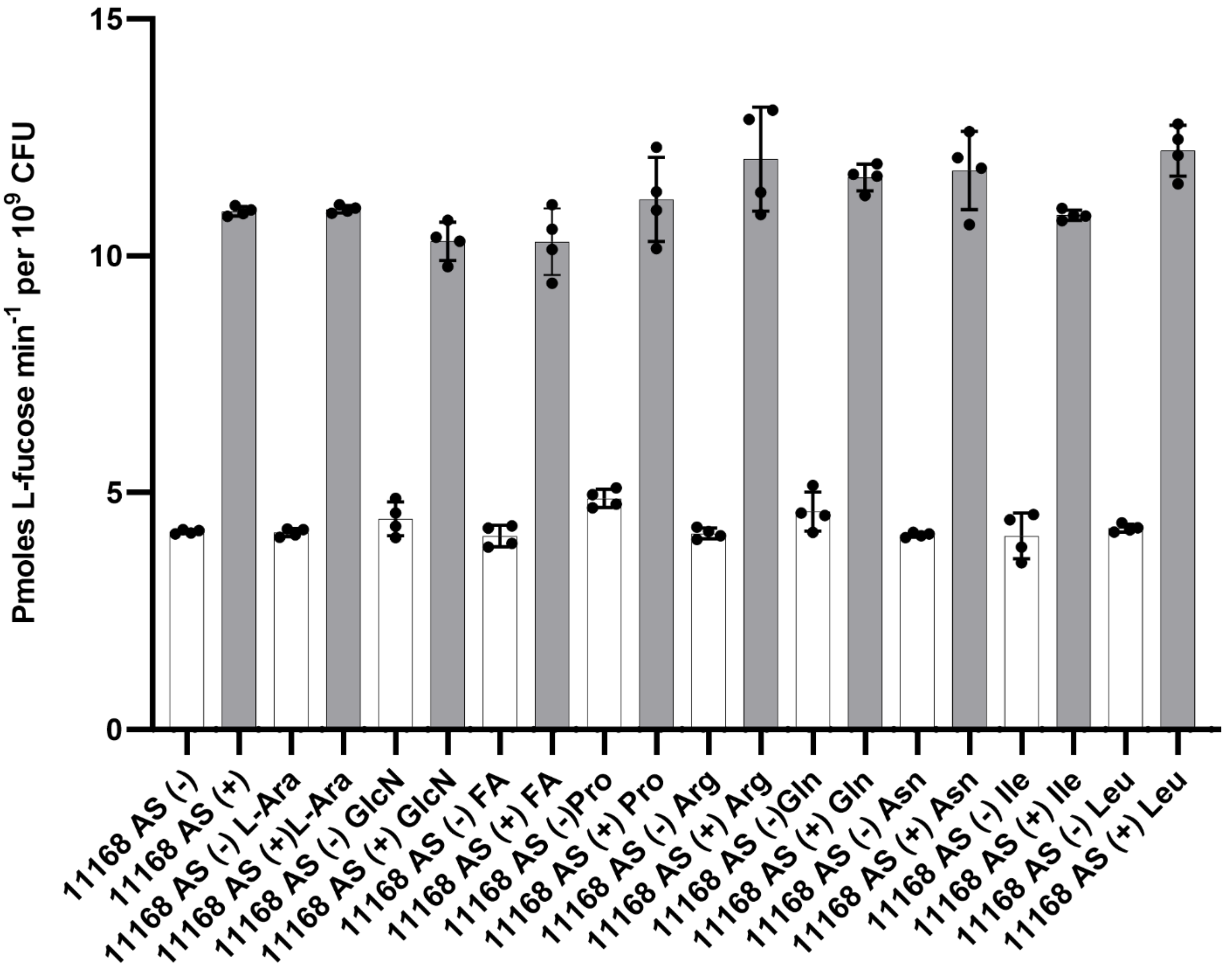

Supplementary Figure 8. **Uptake of L-fucose in the presence of additional carbohydrates by *C. jejuni* 11168 wildtype.** ^{3}H-L-fucose uptake rates are shown in cells grown in the presence (gray) or absence (white) of L-fucose with five-fold excess (50 mM substrate to 10 mM L-fucose) of the indicated alternative carbon sources. Each bar represents the mean value obtained from two independent experiments carried out in duplicate with two technical replicates. Standard deviations are indicated by the error bars and values from individual replicates are overlaid as dots. L-Ara=L-arabinose, GlcN=glucosamine, FA=formic acid, Pro=proline, Arg=arginine, Gln=glutamine, Asn=asparagine, Ile=isoleucine, and Leu=leucine.

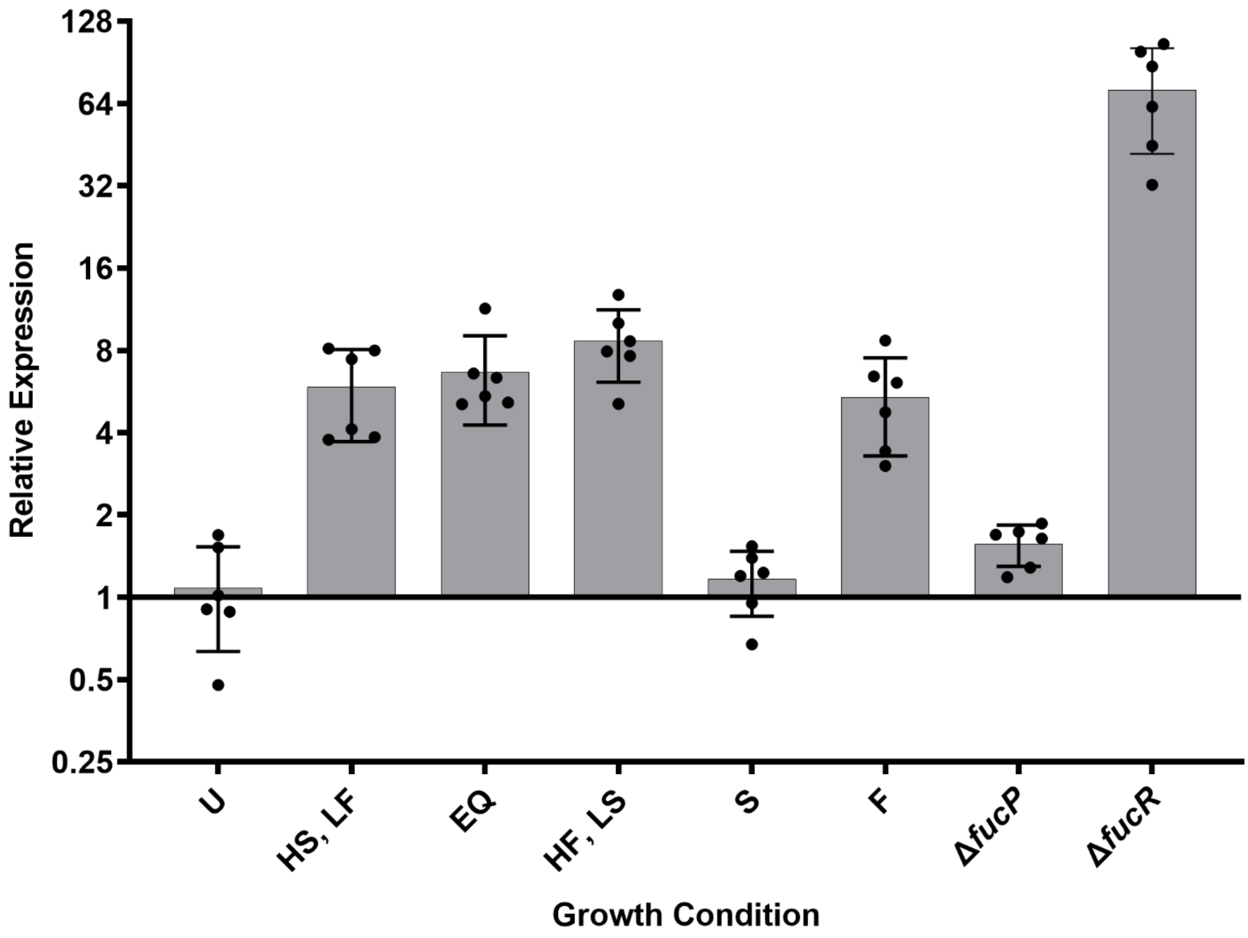

Supplementary Figure 9. **Serine does not transcriptionally regulate *fucP* expression.** Levels of *fucP* expression in the presence of increasing amounts of serine were examined by quantitative RT-PCR. Bars represent means of two biological replicates of three technical replicates of *fucP* expression normalized against 16S expression (normalized to unsupplemented) and error bars show standard error of the mean. Individual values are overlaid as dots. HS, LF=excess serine (50 mM serine, 10 mM fucose), EQ=equimolar (10 mM of each), LS, HF=excess fucose (10 mM fucose, 2 mM serine), serine only (10 mM), fucose only (10 mM), Δ*fucP*=*fucP* mutant, unsupplemented, Δ*fucR*=*fucR* mutant, unsupplemented.

Supplementary Table 1: Significant pathway increases in *B. vulgatus* in the presence of *C. jejuni in vivo*

Pathway ID	Pathway Geometric Mean	Stat Mean	P-value	FDR adjusted P-value	Set size
Metabolic pathways (bvu01100)	9.7E-11	6.438555	9.7E-11	5.82E-09	489
Biosynthesis of secondary metabolites (bvu01110)	1.03E-07	5.28998	1.03E-07	3.08E-06	206
Biosynthesis of antibiotics (bvu01130)	3.64E-06	4.564504	3.64E-06	5.67E-05	165
Biosynthesis of amino acids (bvu01230)	3.78E-06	4.599695	3.78E-06	5.67E-05	110
Microbial metabolism in diverse environments (bvu01120)	8.65E-06	4.393897	8.65E-06	0.000104	119
Carbon metabolism (bvu01200)	2.04E-05	4.249028	2.04E-05	0.000204	72
Fructose and mannose metabolism (bvu00051)	0.000927	3.283636	0.000927	0.007948	27
2-Oxocarboxylic acid metabolism (bvu01210)	0.001563	3.250374	0.001563	0.01172	21
Pentose phosphate pathway (bvu00030)	0.003021	2.881523	0.003021	0.020139	24
Ribosome (bvu03010)	0.004984	2.620968	0.004984	0.028381	63
Amino sugar and nucleotide sugar metabolism (bvu00520)	0.005203	2.632721	0.005203	0.028381	40
Pyruvate metabolism (bvu00620)	0.006938	2.593216	0.006938	0.034691	26

Aminoacyl-tRNA biosynthesis (bvu00970)	0.008073	2.428616	0.008073	0.037261	91
Methane metabolism (bvu00680)	0.011644	2.350691	0.011644	0.046993	23
Purine metabolism (bvu00230)	0.012159	2.288351	0.012159	0.046993	55
Lysine biosynthesis (bvu00300)	0.012531	2.467279	0.012531	0.046993	15

Supplementary Table 2. UniProt IDs for proteins discussed in this publication (in order of appearance)

**Protein Name**	**UniProt ID**
FucX (Cj0485)	Q0PB28_CAMJE
FabG (BmulJ_04919)	A0A0H3KNE7_BURM1
Cj0480c	Q0PB33_CAMJE
Cj0481	Q0PB32_CAMJE
Cj0483	Q0PB30_CAMJE
Cj0484	Q0PB29_CAMJE
Cj0486	Q0PB27_CAMJE
Cj0489	Q0PB24_CAMJE
Cj0487	Q0PB26_CAMJE
Cj0488	Q0PB25_CAMJE

Supplementary Table 3. Oligonucleotides used in this study

Name	Type	Purpose	Sequence (5' – 3')
Cj0485NdeIF	Forward primer	Cj0485 expression construct	GGTCTTCATATGGATTTAAAAATTAAAAATAAGG
Cj0485XhoIR	Reverse primer	Cj0485 expression construct with N-terminal His-tag	CAGTCTCGAGTTACTCCTTAGTTTTCATC
Cj0485XhoIRc	Reverse primer	Cj0485 expression construct with C-terminal His-tag	AATGCTCGAGGTTTTCATCCCAATTTAATGCTC
cj0489newEcoRI-F	Forward primer	*Δcj0489* construct (*cj0489*)	CTAAGAATTCGGATGAAACAAGAATATAAAG
cj0489newXhoI-R	Reverse primer	*Δcj0489* construct (*cj0489*)	TGGACTCGAGCGAGATTTACTTCTAATAAAG
Kan-StyI-F	Forward primer	*Δcj0489* construct (kanamycin cassette)	ATATATATCCTTGGCCATATTTAAAAAGCTACCAAGACG
Kan-StyI-R	Reverse primer	*Δcj0489* construct (kanamycin cassette)	ATATATATCCTTGGAGCTTTTTAGACATCTAAATCTAGG
16s-RT-For	Forward primer	qRT-PCR (*16S* control)	GCGCAACCCACGTATTTAGTTGCT
16s-RT-Rev	Reverse primer	qRT-PCR (*16S* control)	ATGACTTGACGTCGTCCACACCTT
cj0486-RT-F	Forward primer	qRT-PCR (*cj0486*)	GGGAGTAAGCTATGGACTTATTGATGTG
cj0486-RT-R	Reverse primer	qRT-PCR (*cj0486*)	AGCCACTTTCATGCTGGCTAAT
