# Supplementary material for: The gastrointestinal pathogen Campylobacter jejuni metabolizes sugars with potential help from commensal Bacteroides vulgatus
Source: Commun Biol. 2020 Jan 7;3:2. doi: 10.1038/s42003-019-0727-5 (PMC6946681; doi:10.1038/s42003-019-0727-5)
Supplement: Supplementary file 2 — Description of Additional Supplementary Files [file 42003_2019_727_MOESM2_ESM.docx]

**Description of additional supplementary files**

Supplementary Data 1 contains an Excel spreadsheet with the source data underlying Figures 1a-b, 3a-c, and 4a-b and Supplementary Figures 2, 3, 4, 6, 7, 8, and 9. Supplementary Data 2 contains spectral data for Supplementary Figures 1 and 5. Supplementary Data 3 contains the PDB validation report for FucX. Supplementary Data 4 contains the PDB validation report for the FucX with NADPH complex.
