# Supplementary material for: The gastrointestinal pathogen Campylobacter jejuni metabolizes sugars with potential help from commensal Bacteroides vulgatus
Source: Commun Biol. 2020 Jan 7;3:2. doi: 10.1038/s42003-019-0727-5 (PMC6946681; doi:10.1038/s42003-019-0727-5)
Supplement: Supplementary file 4 — Reporting Summary [file 42003_2019_727_MOESM4_ESM.pdf]

nature research

Corresponding author(s): Christine Szymanski

Last updated by author(s): Nov 17, 2019

Reporting Summary

Nature Research wishes to improve the reproducibility of the work that we publish. This form provides structure for consistency and transparency in reporting. For further information on Nature Research policies, see Authors & Referees and the Editorial Policy Checklist .

Statistics

For all statistical analyses, confirm that the following items are present in the figure legend, table legend, main text, or Methods section.

n/a	Confirmed	
☐	☒	The exact sample size (*n*) for each experimental group/condition, given as a discrete number and unit of measurement
☐	☒	A statement on whether measurements were taken from distinct samples or whether the same sample was measured repeatedly
☐	☒	The statistical test(s) used AND whether they are one- or two-sided<br>*Only common tests should be described solely by name; describe more complex techniques in the Methods section.*
☒	☐	A description of all covariates tested
☐	☒	A description of any assumptions or corrections, such as tests of normality and adjustment for multiple comparisons
☐	☒	A full description of the statistical parameters including central tendency (e.g. means) or other basic estimates (e.g. regression coefficient) AND variation (e.g. standard deviation) or associated estimates of uncertainty (e.g. confidence intervals)
☐	☒	For null hypothesis testing, the test statistic (e.g. *F*, *t*, *r*) with confidence intervals, effect sizes, degrees of freedom and *P* value noted<br>*Give P values as exact values whenever suitable.*
☒	☐	For Bayesian analysis, information on the choice of priors and Markov chain Monte Carlo settings
☒	☐	For hierarchical and complex designs, identification of the appropriate level for tests and full reporting of outcomes
☒	☐	Estimates of effect sizes (e.g. Cohen's *d*, Pearson's *r*), indicating how they were calculated

Our web collection on statistics for biologists contains articles on many of the points above.

Software and code

Policy information about availability of computer code

Data collection	Diffraction data were collected on an ''in-house'' beam comprising a Pilatus 200K 2D detector coupled to a MicroMax-007HF X-ray generator with a VariMaxTM-HF ArcSec Confocal Optical System and an Oxford Cryostream 800. Enzymology was conducted with a SpectraMax M5 plate reader. NMR spectra were acquired on a Bruker Neo 800 MHz spectrometer with a 5 mm HCN cryoprobe using standard Bruker pulse sequences (HSQC: hsqcetgpsisp2, HSQC-TOCSY: hsqcetgpml, HMBC: hmbclpndqf). Biolog data were collected using a OmniLog PM system. qRT-PCR data was collected with a Bio-Rad C1000 TouchTM CFX96TM Real-Time System.
Data analysis	Crystallogrpahy data were analyzed using HKL2000, SCALEPACK2MTZ,PHASER, BUCCANEER, COOT, phenix.refine, FINDWATERS, and MOLPROBITY. Michaelis-Menten parameters for enzymology were determined using GraphPad Prism. Statistical tests for growth, uptake, and fucosidase experiments were determined using Microsoft Excel. NMR data were processed using Mestrelab MNOVA software. qRT-PCR data was analyzed with Bio-Rad CFX Manager™ Software 3.1.RNAseq data were analyzed using Bowtie2 and HTSeq.

For manuscripts utilizing custom algorithms or software that are central to the research but not yet described in published literature, software must be made available to editors/reviewers. We strongly encourage code deposition in a community repository (e.g. GitHub). See the Nature Research guidelines for submitting code & software for further information.

Data

Policy information about availability of data

All manuscripts must include a data availability statement. This statement should provide the following information, where applicable:

- Accession codes, unique identifiers, or web links for publicly available datasets
- A list of figures that have associated raw data
- A description of any restrictions on data availability

All relevant data are available from the corresponding author. The source data underlying Figures 1a-b, 3a-c, and 4a-b and Supplementary Figures 2, 3, 4, 6, 7, 8, and 9 are provided as a Source Data file. The spectral data for Supplementary Figures 1 and 5 are provided as a PDF file. The RNAseq data has been deposited to the NCBI SRA archive under accession number PRJNA514712. Coordinates and structure factors for FucX and FucX in complex with NADP+ have been deposited with the PDB codes 6DRR and 6DS1, respectively.

Field-specific reporting

Please select the one below that is the best fit for your research. If you are not sure, read the appropriate sections before making your selection.

- [x] Life sciences
- [ ] Behavioural & social sciences
- [ ] Ecological, evolutionary & environmental sciences

For a reference copy of the document with all sections, see nature.com/documents/nr-reporting-summary-flat.pdf

Life sciences study design

All studies must disclose on these points even when the disclosure is negative.

Sample size	This study did not involve collecting samples.
Data exclusions	Data were excluded if a control in the replicate failed to show the expected phenotype.
Replication	Experiments were reproduced at least once each and additional replicates were added as needed to observe statistical significance.
Randomization	Randomization was not relevant to this study since we were not collecting samples, but rather examining specific lab strains and isogenic mutants.
Blinding	Blinding was not used in this study since no subjective data was collected.

Reporting for specific materials, systems and methods

We require information from authors about some types of materials, experimental systems and methods used in many studies. Here, indicate whether each material, system or method listed is relevant to your study. If you are not sure if a list item applies to your research, read the appropriate section before selecting a response.

Materials & experimental systems

n/a	Involved in the study	
x		Antibodies
x		Eukaryotic cell lines
x		Palaeontology
	x	Animals and other organisms
x		Human research participants
x		Clinical data

Methods

n/a	Involved in the study	
x		ChIP-seq
x		Flow cytometry
x		MRI-based neuroimaging

Animals and other organisms

Policy information about studies involving animals; ARRIVE guidelines recommended for reporting animal research

Laboratory animals	female Swiss Webster germ-free mice
Wild animals	The study did not involve wild animals.
Field-collected samples	The study did not involve field-collected samples.
Ethics oversight	Ethics for this protocol were overseen by the Institutional Animal Care and Use Committee of the University of Ottawa (Animal Care protocol BMI-2347)

Note that full information on the approval of the study protocol must also be provided in the manuscript.
